# Comparison of DNA extraction methods for detecting African swine fever virus in feed and environmental samples

**DOI:** 10.3389/fvets.2025.1675115

**Published:** 2025-10-28

**Authors:** Taeyong Kwon, Jordan T. Gebhardt, Eu Lim Lyoo, Cassandra K. Jones, Jessie D. Trujillo, Natasha N. Gaudreault, Juergen A. Richt

**Affiliations:** ^1^Department of Diagnostic Medicine/Pathobiology, College of Veterinary Medicine, Kansas State University, Manhattan, KS, United States; ^2^Department of Animal Sciences and Industry, College of Agriculture, Kansas State University, Manhattan, KS, United States

**Keywords:** African swine fever, ASFV, DNA extraction, environmental sample, feed, PCR, surface

## Abstract

African swine fever (ASF) is a fatal viral disease of domestic and wild pigs, with mortality rates reaching up to 100%. In the absence of a safe and effective vaccine in non-endemic countries, it is imperative for the swine industry to implement enhanced biosecurity measures to control and prevent ASF outbreaks. Early detection is a key strategy to prevent the introduction of virus (ASFV) into naïve herds and confine the outbreak sites. Despite advanced modern technology for better diagnostics, knowledge regarding ASFV DNA detection in environmental samples is limited. Therefore, this study aimed to compare four DNA extraction methods for ASFV-contaminated feed and environmental samples: two magnetic bead-based extractions, one column-based PowerSoil Pro extraction, and one point-of-care M1 extraction. Three sets of samples were attained from our sample inventory saved from previous ASFV experiments: (1) surface samples contaminated with ASFV and different types of organic matter, (2) ASFV-contaminated feed and feed ingredients, and (3) feed mill surface samples collected during manufacturing of feed inoculated with ASFV. After DNA extraction, quantitative PCR was performed under identical conditions for all samples. ASFV DNA was detected by all four different extraction methods in the first two sets of sample collections. In these samples, significantly lower Cq values (*p* < 0.05) were detected in two magnetic bead-based extractions compared to the column-based PowerSoil Pro and point-of-care M1 extractions. Similarly, better DNA detection was observed using magnetic bead extractions in the feed mill surface samples. We conclude that all extraction methods evaluated in this study can be used for ASFV DNA detection in feed and environmental samples and higher sensitivity was observed using magnetic bead-based extraction which was also able to detect ASFV DNA in feed mill surface samples collected during manufacturing of ASFV-inoculated feed.

## 1 Introduction

African swine fever (ASF) is a contagious and highly fatal disease in domestic pigs and wild boars with up to 100% mortality for acute cases. Its causative agent, African swine fever virus (ASFV), is the only species in the genus *Asfivirus*, Family *Asfarviridae*. It is a large, icosahedral, double-stranded DNA virus with a linear genome of 170–190 kb in length. The genome contains more than 170 genes, and the structure and function of the majority of viral proteins remain unclear.

Polymerase chain reaction (PCR) is one of the fastest and most sensitive laboratory assays for detecting genetic materials in clinical samples. Thus, conventional and real-time qPCR (semi-quantitative) are recommended diagnostic techniques for the identification of ASFV in samples (1). Unlike virus isolation, a PCR assay can be performed in a BSL-2 setting and can utilize a high throughput automated system to accommodate a large number of samples with relatively low cross-contamination. A variety of DNA extractions and PCR assays have been established for over several decades for better detection of ASFV, with improved sensitivity and specificity (2–4). The classical approach uses phenol:chloroform extraction to purify ASFV DNA, however, currently, a number of DNA extraction kits are commercially available for animal disease diagnostics. Spin column-based nucleic acid purification relies on the binding of nucleic acids to a silica membrane. More recently with the advance of molecular diagnostics, magnetic bead-based nucleic acid extraction methods became a more common diagnostic tool as it provides an automated high-throughput system with relatively low cross-contamination issues. In contrast to the laboratory settings which mainly use column- and magnetic bead-based extraction methods, point-of-care (POC) testing allows the detection of ASFV under field conditions without sending the samples to diagnostic laboratories. This increased flexibility is a substantial advantage especially when rapid response efforts are needed to help control the spread of ASFV.

Despite the geographic expansion and genetic diversification of ASFV, the current countermeasures rely on strict biosecurity due to the fact that no commercial vaccine is available in non-endemic countries. The main concept of biosecurity is to break down transmission chains by identifying the agents of concern and inactivating them in an appropriate manner (5). Environmental samples are useful for disease surveillance and provide the basis for biosecurity implementation in endemic areas (6). In addition, environmental surveillance can allow pathogen detection at an early stage of its introduction to pathogen-free areas, further enabling preventive measures to confine the pathogen. Despite the increasing threat of ASFV to become introduced to free areas, current protocols for detecting ASFV have been mainly validated and optimized for clinical samples, such as whole blood and tissue samples. Limited research has focused on evaluating diagnostic methodologies using different sample matrices. Therefore, this study aimed to compare different DNA extraction methods in the context of feed and environmental samples.

## 2 Materials and methods

### 2.1 Ethics statement and viruses

All experiments were approved under the Kansas State University (KSU) Institutional Biosafety Committee (IBC, Protocol #1600) and performed in a biosafety level-3 laboratory in the Biosecurity Research Institute at KSU. Whole blood was used to generate ASFV-contaminated environmental samples and was collected from Georgia07- or Armenia07-infected pigs from a previously conducted animal study and stored at −80 °C.

### 2.2 Feed and environmental samples

Three different sets of samples were generated in our previous studies and retrieved from our inventory (stored at −80 °C): (1) ASFV-contaminated surface samples (7); (2) ASFV-contaminated feed and feed ingredients (8); and (3) feed mill surface samples during ASFV-contaminated feed manufacturing (9).

Briefly, 100 μL (titer: 1.36 × 10^8^ TCID_50_/mL) of blood from Georgia07-infected pigs were mixed with 5 mL of PBS or 2.5 mL of phosphate-buffered saline (PBS) and 2.5 g of four different organic contaminants: soil, swine feces, feed dust and mixture of soil, swine feces and feed dust. ASFV-spiked PBS or organic contaminants were inoculated on 10 × 10 cm stainless steel surface (*n* = 3 per each organic contaminant). The contaminated surfaces were swabbed by two different sampling devices: a premoistened cotton gauze (Dynarex Corporation, Orangeburg, NY, USA) with 5 mL of PBS or a premoistened sponge stick (Cat. #SSL100, 3 M, MN, United States) with 10 mL of DNA/RNA shield (Zymo Research Irvine, CA, USA). The cotton gauze sample was placed back to a 50 mL conical tube, and 5 mL of PBS was added. The sponge stick was placed back to a plastic bag, and an additional 5 mL of PBS was added to the samples derived from feed dust and mixture-contaminated surfaces. After vortexing or massaging, supernatant was transferred into a cryovial and stored at −80 °C.

ASFV-contaminated feed and feed ingredients were manufactured at a BSL-3Ag animal room. A total of 530 mL of Armenia07 (virus titer: 2.7 × 10^6^ TCID_50_/mL) was mixed with 4.7 kg of complete feed in a 5 kg stainless steel mixer (Cabela’s Inc., Sidney, NE). This mixture was subsequently mixed with an additional 20 kg complete feed to make a final concentration of 5.6 × 10^4^ TCID_50_/g, conveyed and discharged into the bags (8). Ten grams of completed feed samples were collected using two ‘x’ patterns (10). To manufacture ASFV-contaminated feed ingredients, 100 mL of Armenia07 (virus titer: 6.5 × 10^5^ TCID_50_/mL) was mixed with 1 kg of soybean meal or spray-dried plasma in a manual hand mixer, resulting in a final concentration of 6.5 × 10^4^ TCID_50_/g. Ten grams of ASFV-contaminated soybean meal and spray-dried plasma were collected in a 50-mL conical tube. After adding 35 mL of PBS, the tube was incubated overnight at 4 °C. Supernatant was transferred into a cryovial and stored at −80 °C.

Feed mill surface samples were collected after ASFV-contaminated complete feed was manufactured. Locations for environmental sampling were six feed contact surfaces, five non-feed contact surfaces less than 1 m from feed location, four non-feed contact surfaces more than 1 m from feed location and three boot soles of researchers (9). The environmental surfaces were swabbed using a premoistened cotton gauze with 5 mL of PBS, and the gauze was placed into a 50-mL conical tube. After adding 20 mL of PBS, the tube was incubated overnight at 4 °C. Supernatant was transferred into a cryovial and stored at −80 °C.

### 2.3 DNA extraction and quantitative PCR (qPCR)

The samples were retrieved from the −80 °C freezer and subjected to four different extractions and qPCR assays at the same time. The feed and environmental samples were thawed and centrifuged at 700×*g* for 5 min to remove the debris. An equal volume of supernatant was mixed with the AL lysis buffer (Qiagen, Germantown, MD, USA) to make AL lysate and incubated at 70 °C for 10 min for efficient lysis. DNA was extracted using four different methods.

Automated magnetic bead-based DNA extraction using taco™ DNA/RNA Extraction Kit (GeneReach, Lexington, MA, USA). A total of 200 μL of AL lysate and 200 μL of molecular grade isopropanol were added into the first column of the deep-well extraction plate that contained 200 μL of Lysis buffer and 50 μL of magnetic beads. The extraction was performed using taco™ mini automated nucleic acid extraction system (GeneReach, Lexington, MA, USA) and underwent four washing steps: two washes with 750 μL of washing buffer A, one wash with 750 μL of washing buffer B, and a final wash with 200 μL of 200 proof molecular grade ethanol. Viral DNA was eluted in 100 μL of elution buffer and transferred to the tube.

Automated magnetic bead-based DNA extraction using MagMAX™ Pathogen RNA/DNA Kit (Applied Biosystems). A total of 200 μL of AL lysate, 20 μL of beads/bead enhancer and 200 μL of molecular grade isopropanol were added into the pre-filled extraction plate. Automated extraction was performed using KingFisher™ Duo Prime Purification System (Thermo Scientific, Waltham, MA, USA). The wash step consists of two washes with 300 μL of W1 buffer, one wash with 450 μL of W2 buffer and one wash with 450 μL of 200 proof molecular grade ethanol. Viral DNA was eluted in 100 μL of Elution buffer and transferred to the tube.

Column extraction using DNeasy PowerSoil Pro kit (Qiagen, Germantown, MD, USA). A total of 200 μL of AL lysis buffer was mixed with 800 μL of Solution CD1, homogenized for 10 min and centrifuged at 15,000×*g* for 1 min. Clarified supernatant was mixed with 200 μL of Solution CD2 and centrifuged at 15,000×*g* for 1 min. Seven hundred microliters of clarified supernatant were mixed with 600 μL of Solution CD3, and mixture was loaded and passed through a spin column twice. The column was washed twice with 500 μL of Solution EA and Solution C5. After removing the residual buffer by centrifugation at 15,000×*g* for 1 min, viral DNA was eluted in 100 μL of Solution C6 and transferred to the tube.

Point-of-care extraction using M1 Sample Prep Cartridge Kit for DNA-HI (Biomeme, Philadelphia, PA, USA). A total of 200 μL of AL lysate was added into the Sample Prep Cartridge containing lysis buffer. DNA was bound to the Sample Prep Column attached to the syringe by repeating 10 pumps. DNA washing was performed by repeating pumps on each section containing a series of wash buffers. After air drying, viral DNA was eluted in 850 μL of elution buffer and transferred to the tube.

Five microliters of viral DNA were mixed with p72-specific primers and probe (11) in PCR mastermix (PerfeCTa^®^ FastMix^®^ II, Quanta Biosciences; Gaithersburg, MD, USA) (12). PCR reaction was performed in duplicate wells using the CFX 96 PCR machines. The sample was considered positive if detected in duplicate wells and suspect-positive if detected in one of two replicates in PCR.

### 2.4 Statistical analysis

Analysis of variance with Tukey’s adjustment using quantification cycle (Cq) values was performed for ASFV-contaminated surface and feed samples to control Type I error rate, and results were considered significant at *p* < 0.05. Visual assessment of studentized residual plots was performed to evaluate model assumptions which appeared to be reasonably met. Fisher’s exact test was used to compare the proportion of positive samples (including suspect-positive) between DNA extraction techniques in feed mill surface samples, and results were considered significant at *p* < 0.05.

## 3 Results

**Figure 1:**
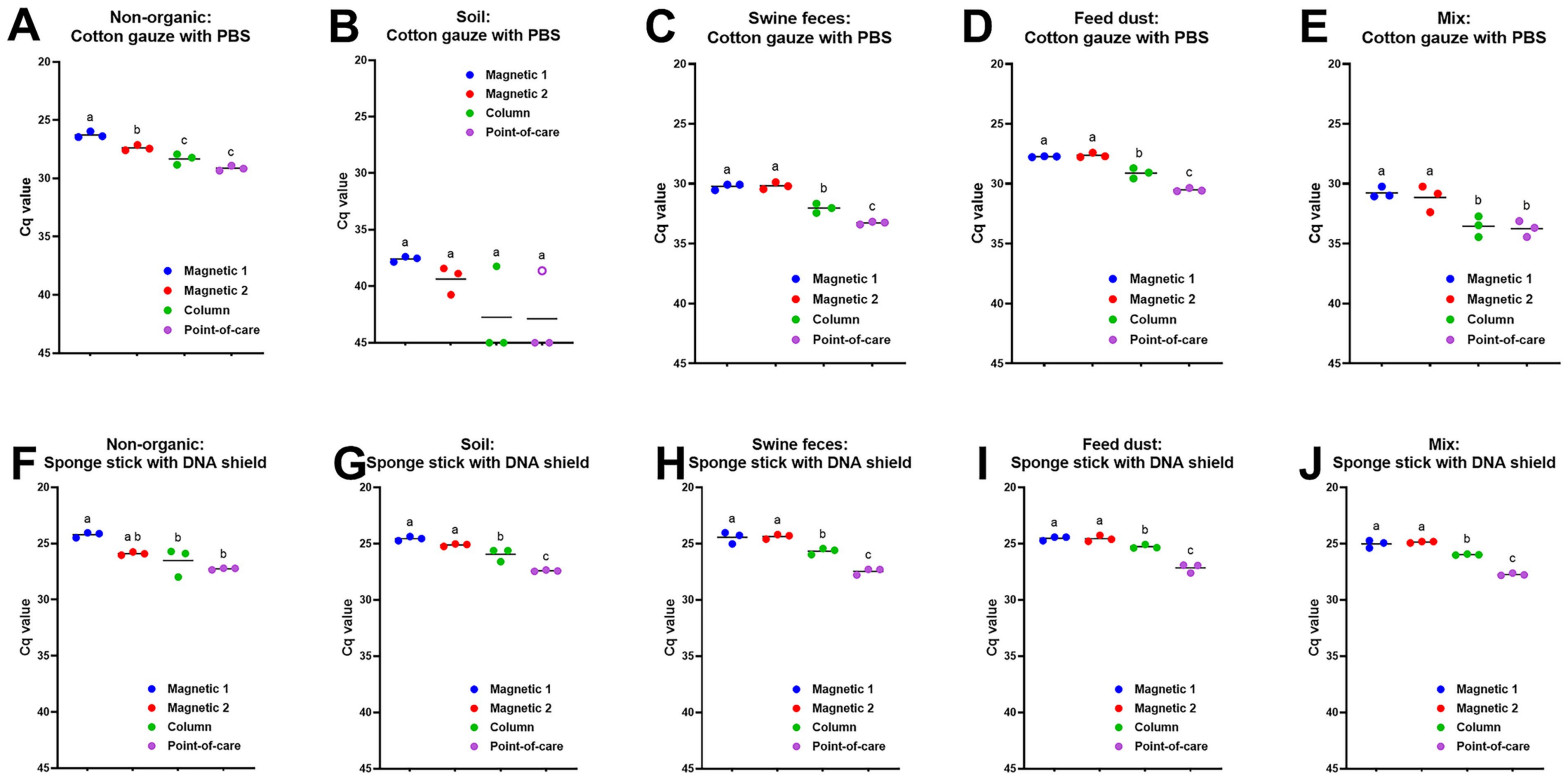
ASFV DNA detection in ASFV-contaminated surface samples using four different DNA extraction protocols. Stainless steel surfaces in triplicate were contaminated with a mixture of 100 μL of ASFV-infected blood and 5 mL of PBS **(A, F)** or 2.5 mL of PBS and 2.5 g of four different organic contaminants: soil **(B, G)**, swine feces **(C, H)**, feed dust **(D, I)**, and their mixture **(E, J)**. The surfaces were swabbed using two different methods: the premoistened cotton gauze **(A–E)** or the sponge stick with DNA/RNA shield **(F–J)**. Viral DNA was extracted from supernatants using four different methods. Magnetic 1: Magnetic bead-based automated DNA extraction using taco™ DNA/RNA Extraction Kit; Magnetic 2: Magnetic bead-based automated DNA extraction using MagMAX™ Pathogen RNA/DNA Kit; Column: Column extraction using DNeasy PowerSoil Pro kit; Point-of-care: Point-of-care extraction using M1 Sample Prep Cartridge Kit for DNA-HI. p72 qPCR was performed in duplicate wells under identical conditions. An open circle represents suspect-positive that was a single positive out of duplicate wells. Cq values were used for ANOVA and subsequent Tukey’s adjustment. Statistical differences (*p* < 0.05) were shown by different letters.

To compare the effect of DNA extraction methods on ASFV detection in environmental samples, three sets of the samples were retrieved from our inventory. Viral DNA was extracted using four different DNA isolation protocols and amplified under identical qPCR conditions. First, we used environmental samples collected from ASFV-contaminated stainless steel. When samples were collected from surfaces with no organic material contamination using the premoistened gauze sampling device, Cq values using both magnetic bead-based automated extraction methods were significantly lower (*p* < 0.05) than those using the column-based PowerSoil Pro and point-of-care M1 extraction methods (Figure 1A). In addition, we observed a significant difference between the two magnetic bead-based extraction systems with those samples, with the taco DNA/RNA Extraction Kit having lower Cq values compared to the MagMAX Pathogen RNA/DNA kit (*p* < 0.05) (Figure 1A). High Cq values were obtained in environmental samples collected from surfaces contaminated with soil using the premoistened gauze sampling device, but the magnetic bead-based extraction resulted in lower Cq values, when compared to the column-based PowerSoil Pro and point-of-care M1 extractions (Figure 1B). For the environmental samples contaminated with swine feces, feed dust, or a mixture of the above using the premoistened cotton gauze sampling device, significantly lower (*p* < 0.05) Cq values were obtained using the magnetic bead-based automated DNA extractions than using the column-based PowerSoil Pro or point-of-care M1 extractions (Figures 1C–E). The column-based PowerSoil Pro extraction provided lower Cq values (*p* < 0.05) than the point-of-care M1 extraction for swine feces or feed dust-contaminated surface samples swabbed using cotton gauze (Figures 1C,D). We observed a similar pattern of lower Cq values (*p* < 0.05) using the magnetic bead-based extraction in these environmental samples collected using the pre-moistened sponge stick with DNA/RNA shield (Figures 1F–J). In addition, lower Cq values (*p* < 0.05) were obtained in environment samples collected from surfaces contaminated with soil, swine feces, feed dust, or a mixture thereof using the premoistened sponge stick with DNA/RNA shield when using the column-based PowerSoil Pro extraction, when compared to the point-of-care M1 extraction (Figures 1G–J).

**Figure 2:**
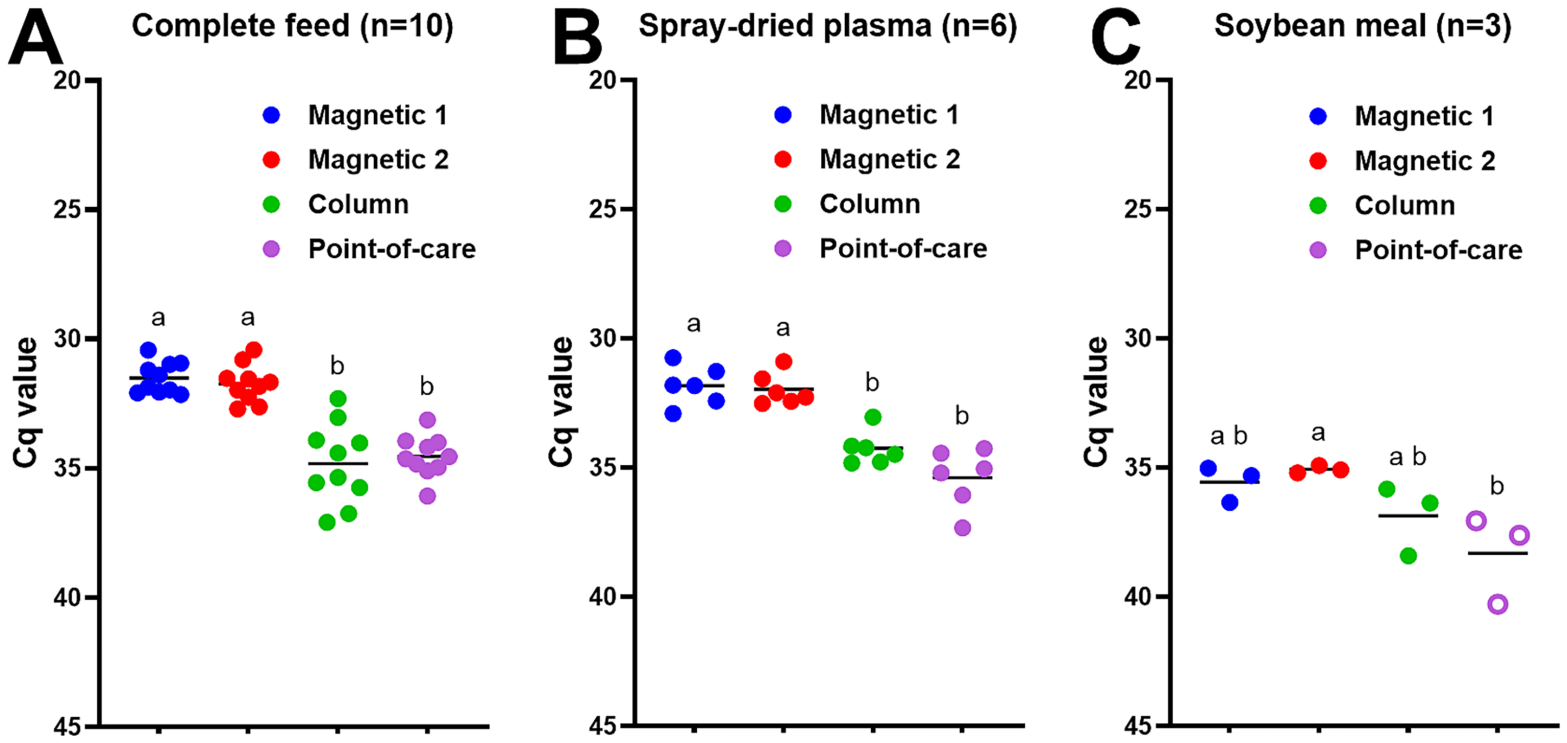
ASFV DNA detection in ASFV-contaminated feed and feed ingredients using four different DNA extraction protocols: **(A)** complete feed (*n* = 10), **(B)** spray-dried plasma (*n* = 6), and **(C)** soybean meal (*n* = 3). ASFV-contaminated feed and feed ingredients were manufactured at the concentration of 5.6 × 10^4^ TCID_50_/g for complete feed and 6.5 × 10^4^ TCID_50_/g for spray-dried plasma and soybean meal, respectively at the BSL-3 facility. Ten grams of feed or feed ingredients were mixed with 35 mL of PBS, and supernatant was collected. Viral DNA was extracted from the supernatant using four different methods. Magnetic 1: Magnetic bead-based automated DNA extraction using taco™ DNA/RNA Extraction Kit; Magnetic 2: Magnetic bead-based automated DNA extraction using MagMAX™ Pathogen RNA/DNA Kit; Column: Column extraction using DNeasy PowerSoil Pro kit; Point-of-care: Point-of-care extraction using M1 Sample Prep Cartridge Kit for DNA-HI. p72 qPCR was performed in duplicate wells under identical conditions. An open circle represents suspect-positive that was a single positive out of duplicate wells. Cq values were used for ANOVA and subsequent Tukey’s adjustment. Statistical differences (*p* < 0.05) were shown by different letters.

Next, we evaluated different DNA extraction methods for ASFV-contaminated feed and feed ingredients. Small batches of ASFV-contaminated complete feed, soybean meals, and spray-dried plasma were manufactured, collected, processed, and stored at −80 °C as described in Materials and Methods. Both magnetic bead-based extraction protocols yielded lower Cq values (*p* < 0.05) than the column-based PowerSoil Pro or point-of-care M1 protocols in all ASFV-contaminated complete feed samples (Figure 2A). There was no significant difference in Cq values among the two magnetic bead extraction methods (Figure 2A). We obtained similar results for ASFV-contaminated spray-dried plasma (Figure 2B). For the ASFV-contaminated soybean meal samples, the magnetic bead-based MagMax extraction showed better sensitivity (*p* < 0.05) than the point-of-care M1 extraction method (Figure 2C).

**Figure 3:**
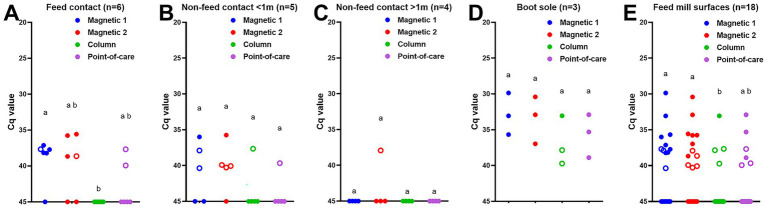
ASFV DNA detection in feed mill surface samples using four different DNA extraction protocols: **(A)** feed contact surfaces, **(B)** non-feed contact surfaces less than 1 m from feed location, **(C)** non-feed contact surfaces more than 1 m from feed location, **(D)** boot soles of researchers, and **(E)** all surface samples. Surface samples were collected using premoistened cotton gauze after ASFV-contaminated complete feed was manufactured. The supernatant was collected after adding 20 mL of PBS. Viral DNA was extracted from the supernatant using four different methods. Magnetic 1: Magnetic bead-based automated DNA extraction using taco™ DNA/RNA Extraction Kit; Magnetic 2: Magnetic bead-based automated DNA extraction using MagMAX™ Pathogen RNA/DNA Kit; Column: Column extraction using DNeasy PowerSoil Pro kit; Point-of-care: Point-of-care extraction using M1 Sample Prep Cartridge Kit for DNA-HI. p72 qPCR was performed in duplicate wells under identical conditions. An open circle represents suspect-positive that was a single positive out of duplicate wells. The significant difference in the proportion of positive samples was determined Fisher’s exact test, and statistical differences (*p* < 0.05) were shown by different letters.

Lastly, the environmental samples from feed mill surfaces were tested with the different DNA extraction methods. ASFV DNA was detected in five out of six feed contact surfaces: four positive and one suspect-positive out of six surface samples using magnetic bead-based taco extraction, three positive and one suspect-positive with the magnetic bead-based MagMax extraction, and two suspect-positive with the point-of-care M1 extraction, however none of them (0/6) were positive with the column-based PowerSoil Pro extraction method (Figure 3A). The proportion of positive/suspect-positive samples using magnetic bead-based taco extraction was significantly higher (*p* < 0.05) than that by the column-based PowerSoil Pro extraction (Figure 3A). Four of five non-feed contact surfaces less than 1 m from the feed mill location were positive/suspect-positive by at least one protocol: ASFV DNA was detected in three samples (one positive and two suspect-positive) by the magnetic bead-based taco extraction, four (one positive and three suspect-positive) by the magnetic bead-based MagMax extraction, and one (suspect-positive) by the column-based PowerSoil Pro and point-of-care M1 extractions (Figure 3B). In contrast, only one non-feed contact surface sample more than 1 m from the feed mill location was suspect-positive using the magnetic bead-based MagMax extraction method (Figure 3C). All three boot soles were positive by two magnetic bead-based and point-of-care M1 extractions, one was positive and two were suspect-positive by the column-based PowerSoil Pro extraction (Figure 3D). In addition, lower Cq values were observed using the magnetic bead-based extraction methods compared to the other two methods (Figure 3D). Across all sample locations (Figure 3E), the proportions of positive/suspect-positive samples using the two magnetic bead-based extraction methods were significantly higher (*p* < 0.05) than using the column-based PowerSoil Pro extraction: eight positive and three suspect-positive out of 18 samples for the magnetic bead-based taco extraction, seven positive and five suspect-positive for the magnetic bead-MagMax extraction, one positive and three suspect-positive for the column-based PowerSoil Pro extraction, and three positive and three suspect-positive for the point-of-care M1 extraction.

## 4 Discussion

The geographical expansion of ASFV and continuous ASF outbreaks in Europe and Asia pose significant threats to the global pork industry. The recent introduction of genotype II ASFV into the island of Hispaniola in the Caribbean has triggered significant concerns for the Americas (13). Due to no effective and safe vaccine available in non-endemic countries, rapid and accurate detection is a key element for successful prevention and control of ASF Molecular testing offers a rapid turnaround time and high diagnostic accuracy for a variety of infectious diseases in the veterinary field. Nucleic acid extraction and subsequent real-time PCR are the recommended procedures for ASFV diagnostics and various PCR tests have been validated with clinical samples (1). Despite extensive environmental contamination with ASFV from infected pigs and its high stability in environments (14, 15), our understanding of ASFV molecular diagnostics in environmental samples still remains limited. Therefore, the present study aimed to compare different DNA extraction methods for ASFV DNA detection in feed and environmental samples. Three different sets of ASFV-contaminated samples were procured from previous studies (7–9) and subjected to four different DNA extraction protocols: two magnetic bead-based automated extractions, one column-based PowerSoil Pro extraction and one point-of-care M1 extraction. The first set of samples were surface samples contaminated with high titers of ASFV and in addition, with different types of organic matter (7). For the second set of samples, ASFV was directly mixed with complete feed or two feed ingredients, and the samples were collected directly from a single batch of contaminated complete feed or feed ingredients (8). The third set of samples was feed mill environmental samples which were collected from various surfaces, which included feed contact areas and non-feed contact areas, during manufacturing of complete feed inoculated with ASFV (9). Thus, the level of ASFV DNA contamination varied significantly in these types of environmental samples; even some non-feed contact surfaces were potentially contaminated with ASFV DNA in indirect ways via feed dust or aerosols. The four DNA extraction methods compared here were shown to be suitable for ASFV DNA extraction. However, the magnetic bead-based extraction methods resulted in lower Cq values, specifically for the first two sets of samples that were directly spiked with high titer ASFV. Similarly, the ASFV detection rate in feed mill surface samples was higher with magnetic bead-based extraction methods even at lower ASFV contamination levels present during manufacturing of ASFV-inoculated feed.

The key to successful isolation of viral nucleic acids for laboratory diagnosis is to ensure that intact DNA/RNA is released and purified from virions or infected cells/tissues with efficient removal of potential PCR inhibitors and nucleases during extraction procedures. A variety of commercial DNA isolation kits have been tested for detecting ASFV DNA in clinical samples, such as blood, tissues, or oral fluids (2, 4, 16). In the present study, the selection of the four DNA extraction kits was dependent on their potential use for rapid ASFV detection in environmental samples where organic contaminants potentially inhibit the subsequent PCR reaction. We chose two different magnetic bead-based extraction methods based on their common use in ASFV diagnostic laboratories. The modified protocol of the taco™ DNA/RNA Extraction Kit has been used in our laboratory for over a decade and was validated for multiple clinical samples (12). In addition, its capability to detect ASFV DNA in environmental samples has been shown in our previous studies (7, 17, 18). The other magnetic bead-based extraction method, the MagMAX™ Pathogen RNA/DNA Kit, has been widely used for ASFV diagnostics in North America (19, 20) and is listed as a National Animal Health Laboratory Network (NAHLN) standard operating procedure (21). The DNeasy PowerSoil Pro kit was selected for the solid-phase extraction using silica gel spin columns due to its common use to characterize microbial genetic composition in soils (22, 23), although other commercial column-based kits, such as QIAamp Viral RNA mini kit, have been used for clinical samples in ASFV diagnostics (24–27). Our selection was based on its designated use for soil samples and its capability of PCR inhibitor removal. The fourth method was the point-of-care (POC) M1 Sample Prep Cartridge Kit, because it was tested with clinical samples for ASFV diagnostics in a previous study (28), and we wanted to determine whether point-of care diagnostics of feed and environmental samples is feasible. We found that both magnetic bead-based extraction methods resulted in lower Cq values in feed and environmental samples contaminated with high titers of ASFV and better qPCR positive rates in feed mill surface samples. The DNeasy PowerSoil kit is designated for efficient genomic DNA isolation from soil bacteria and fungi and optimized for subsequent analysis of the relatively large DNA fragments, such as 16S rRNA (22, 23). In addition, spin columns usually exclude fragments of less than 200 nucleotides. Since ASFV qPCR diagnostics uses the amplification of rather small pieces of viral DNA (11), this may explain the better performance of the magnetic bead-based extractions for detecting ASFV DNA in feed and environmental samples. In addition, the ability of PCR inhibitor removal, nucleic acid binding efficiency, and lysis effectiveness may influence the increased performance of magnetic bead-based DNA extraction.

In general, POC molecular diagnostics enables on-site analysis in the field for rapid detection of infectious diseases. Previous studies tested a variety of POC methods for extracting and amplifying ASFV DNA with different types of clinical samples (24, 28–30). The major obstacle of POC testing is limited availability of laboratory equipment in the field, most of which are too heavy to transport to the site for testing and require electricity connection to operate the system. To overcome these limitations, crude DNA is occasionally extracted by heat-treatment from clinical samples and then subjected to the POC PCR reaction on a portable device (29, 30). Using an advanced system, DNA is extracted and purified utilizing a filtration-based method in which nucleic acids selectively bind to the silica membrane inside a syringe as employed by the M1 Sample Prep Cartridge Kit. Subsequent washes with syringes through a sequence of specially formulated buffers yield purified nucleic acids upon elution. This method requires no lab equipment, refrigeration, electricity, alcohol precipitation or phenol-chloroform extraction. It was previously demonstrated that this kit provided comparable performance to the laboratory-based assay on whole blood, serum, meat exudate, tissues and swab samples (28). Our study indicated that viral DNA was also successfully isolated using the POC extraction method, although we observed higher Cq values compared to the other extraction methods. One reason for these results could be that the POC kit uses a much larger volume of elution buffer (850 μL) when compared to the other extraction protocols (100 μL). This results in a lower DNA content per uL eluate and may explain the reduced sensitivity using the POC extraction in our feed and environmental samples. The POC extraction protocol may need to be optimized for better ASFV detection in feed and environmental samples as these sample types contain an unpredictable level of ASFV DNA. It should be noted that the present study evaluated DNA extraction efficiency of different kits under identical, lab-based qPCR conditions; this approach might be suboptimal for the POC extraction protocol which is usually combined with POC PCR detection devices. Therefore, additional studies are needed to determine the performance of integrated POC testing by completing the ASFV PCR reaction using the portable thermocycler developed for the POC kit. Optimized, integrated POC testing would provide valuable opportunities for on-site testing with a rapid turnaround time in low-resource regions or in case of delays or long distances for transporting samples to an approved diagnostic laboratory.

There are several limitations to this study. First of all, the study only used early genotype II strains (Georgia07 and Armenia07) for generating the contaminated samples. Given that ASFV has underwent geographic expansion and genetic diversification, testing contemporary strains would improve the generalizability of our findings to current global events. Secondly, this study did not quantify PCR inhibition in feed and environmental samples that are expected to contain the high levels of PCR inhibitors. Testing internal control genes, such as GAPDH or *β*-actin, would strengthen our overall conclusions. In addition, virus titration would provide additional information on the performance of the different methods, especially for low-contaminated samples. Lastly, the POC extraction method was not optimized in terms of elution volume and use with a portable PCR device. Adjusting the elution buffer volume and/or evaluating the M1 kit with its matched portable device would most likely improve its performance and its potential utility in real-world applications.

In summary, the present study evaluated different DNA extraction methods for detecting ASFV in environmental and feed samples. In samples contaminated with high amounts of ASFV DNA, all methods successfully isolated viral DNA although differences existed between methods with the magnetic bead-based techniques generally performing better. For feed mill surfaces with largely lower ASFV contamination levels, the magnetic bead-based techniques generally performed better than the columns-based PowerSoil Pro or point-of-care M1 methods. It should be noted that we tested only a few selected kits from the wide range of commercially available options using the manufacturers’ recommended protocols that are not optimized for environmental samples. Therefore, ASFV DNA extraction protocols should be selected and optimized based on specific needs, kit availability, time constraints, lab equipment, and biosafety requirements.

## Data Availability

The original contributions presented in the study are included in the article/supplementary material, further inquiries can be directed to the corresponding authors.
